# SMAD4 Protein Expression Is Downregulated in Ileal Epithelial Cells from Patients with Crohn's Disease with Significant Inverse Correlation to Disease Activity

**DOI:** 10.1155/2018/9307848

**Published:** 2018-05-24

**Authors:** Pia Klausen, John Gásdal Karstensen, Mehmet Coskun, Adrian Săftoiu, Peter Vilmann, Jack Bernard Cowland, Lene Buhl Riis

**Affiliations:** ^1^Gastro Unit, Copenhagen University Hospital Herlev and Gentofte, DK-2730 Herlev, Denmark; ^2^Biotech Research and Innovation Centre (BRIC), University of Copenhagen, DK-2200 Copenhagen, Denmark; ^3^Research Center of Gastroenterology and Hepatology, University of Medicine and Pharmacy, 200349 Craiova, Dolj, Romania; ^4^Department of Clinical Genetics, Rigshospitalet, DK-2100 Copenhagen, Denmark; ^5^Department of Pathology, Copenhagen University Hospital Herlev and Gentofte, DK-2730 Herlev, Denmark

## Abstract

**Background:**

Small mothers against decapentaplegic (SMAD)4 and SMAD7 are key regulatory components in the immunosuppressive transforming growth factor- (TGF-) *β* signaling pathway, which is defective in inflammatory bowel disease (IBD). SMAD4 may play an important role in the pathogenesis of IBD as indicated in experimental models of colitis.

**Aims:**

To examine the ileal expression levels of SMAD4 and to correlate these with CD disease activity.

**Methods:**

The material comprised 29 CD patients (13 with active disease, 16 in remission) and 9 asymptomatic patients referred for ileocolonoscopy as part of an adenoma surveillance program serving as controls. Patients were examined with ileocolonoscopy. Corresponding ileal biopsies were obtained for histological analysis and assessment of SMAD4 and SMAD7 protein expression by immunohistochemistry (IHC).

**Results:**

The protein expression of SMAD4 was significantly downregulated in ileal tissue sections from CD patients as compared to healthy controls (*p* < 0.001). Further, luminal SMAD4 expression was inversely correlated with endoscopic (*r*_s_ = −0.315; *p* = 0.05) and histopathological activity (*r*_s_ = −0.40; *p* = 0.013).

**Conclusions:**

The SMAD4 epithelial protein level was markedly downregulated in CD patients and inversely correlated with disease activity. This may contribute to defective mucosal TGF-*β* signaling in active IBD.

## 1. Introduction

The disruption of the colonic epithelial barrier is associated with inflammatory bowel disease (IBD) [1], and achievement of mucosal healing is an important treatment goal [2], since it is associated with sustained steroid-free clinical remission and reduced risk of surgery [3]. Once the epithelial barrier is disrupted, commensal bacteria and their products may cross the intestinal wall [4, 5] and give rise to an inappropriate immune response, which perpetuates chronic inflammation.

A key anti-inflammatory cytokine is transforming growth factor- (TGF-) *β* [6], which is constitutively expressed in the gut by intestinal epithelial cells (IECs), fibroblasts, and lamina propria mononuclear cells (LPMCs) [7]. The TGF-*β* level is elevated in IBD [8], but TGF-*β* signaling has been proposed defective in IBD and thus counteracts the increased TGF-*β* levels [9]. A phase 2 study showed that reduction of the TGF-*β* signaling inhibitor SMAD7 by administration of an antisense oligonucleotide (mongersen) resulted in a significant improvement in Crohn's disease (CD) activity compared with placebo [10, 11]. Mongersen has also been shown to improve Simple Endoscopic Score for Crohn's Disease (SES-CD) [12]. However, a recent phase 3 study has been terminated early due to negative results in an interim analysis. The downstream signaling protein SMAD4, a common SMAD, which is required for full activation of TGF-*β* signaling, is the only mammalian common SMAD and may be equally important in IBD. SMAD4 forms a multi-SMAD complex with phosphorylated and thus activated SMAD2 and SMAD3. This complex translocates to the nucleus and acts as a transcription factor complex on a variety of genes [6]. Of note, *Smad4^+/−^* mice are more prone to develop acute inflammation as seen in a chemically-induced colitis model [13]. Mice carrying a deletion of the *Smad4* gene selectively in T cells develop strong gastrointestinal inflammation and eventually cancer [14]. Hence, SMAD4 and SMAD7 have opposing effects on the TGF-*β* signaling pathway; however, little is currently known about the role of SMAD4 in patients with IBD.

The primary aim of this study was to examine the protein expression levels of SMAD4 in mucosal biopsies obtained from the terminal ileum of patients with CD and to correlate the expression levels with CD activity.

## 2. Materials and Methods

### 2.1. Patients

Patients referred for ileocolonoscopy at the Copenhagen University Hospital Herlev and Gentofte were prospectively enrolled in the study. Twenty-nine patients with CD were included and comprised 16 in remission and 13 with active disease. Active disease was defined using SES-CD score as described by Daperno et al. [15]. The patients were classified as being in endoscopic remission, if SES-CD < 3 in the terminal ileum and colon [15]. Relapse was defined as need for treatment escalation or surgical intervention during the follow-up period.

Further, histopathological changes as described by D'Haens et al. [16] were scored blindly by an experienced pathologist (LBR) and categorized as normal, with chronic inactive inflammation or chronic active inflammation. The control group consisted of 9 asymptomatic patients referred for ileocolonoscopy as part of an adenoma surveillance program, where biopsies were obtained from endoscopically normal terminal ileum and confirmed by histology. Patients below 18 years, pregnant or breastfeeding, and with impaired renal function were excluded from the study. All patients provided written informed consent to participate in the study. The groups were equally distributed in terms of sex, but the individuals in the control group were older than the CD patients. Due to scarce biopsy material, only 11 patients with active disease and 9 patients in remission were analyzed for SMAD7 expression.  Table 1 summarizes the patient characteristics and medication of the CD patients. The study was approved by the Scientific Ethics Committee of the Capital Region of Denmark (September 17, 2012, protocol number H-1-2012-089) and the Danish Data Protection Agency (September 13, 2012, protocol number HEH.750.89-32) and was registered in http://clinicaltrials.gov (NCT01684514). The study protocol conforms to the ethical guidelines of the 1975 Declaration of Helsinki as reflected in a prior approval by the institution's human research committee.

### 2.2. Endoscopy

Ileocolonoscopy was performed with a white light confocal laser endomicroscope (CLE) colonoscope (EC-3837CILK, Pentax Medicals) due to a parallel study on CLE in CD. SES-CD assessment was performed in the terminal ileum and colon [15]. Biopsy specimens were obtained at identical sites in the terminal ileum.

### 2.3. Immunohistochemistry (IHC)

The biopsy specimens were formalin fixed and paraffin-embedded (FFPE). From each biopsy, 3 *μ*m sections were cut and stained with haematoxylin and eosin. The biopsy specimens were assessed in a blinded manner by LBR and scored as described by D'Haens et al. [16]. Sections from the FFPE terminal ileum biopsies were pretreated by deparaffinization, rehydration, and epitope retrieval in one step (high pH) by the use of “Pretreatment Module” (PT-link; Dako, Glostrup, Denmark) according to the manufacturer's instruction. IHC staining was performed using the “Dako Autostainer Link” and with the commercially available kit (EnVision Flex; Dako). Slides were counterstained with Mayers haematoxylin before viewing. The antibodies used were SMAD4 ((B-8) : sc7966, dilution 1 : 1000; Santa Cruz Biotechnology) and SMAD7 ((Z8-B) : sc-101152, dilution 1 : 100; Santa Cruz Biotechnology). Ductal breast carcinoma tissue was used as positive control for SMAD4 and SMAD7. Isotype controls were run in parallel as negative controls. IHC was done in one procedure for all sections to ensure equal staining. The laboratory has been accredited according to the “Danish Healthcare Quality Programme” and participates in NordicQC (Nordic Immunohistochemical Quality Control).

The evaluation of the stained slides was performed by semiquantitative scoring of the intensity of the staining on the villi (luminal) and crypts (basal) for SMAD4. SMAD7 was given one score for the percentage of stained cells in the lamina propria. The SMAD4 immunoreactivity was graded from 0–3 according to the intensity: 0 for absent, 1 for weak, 2 for moderate, and 3 for strong staining. The intensity scoring was performed blindly by LBR.

### 2.4. Statistics

Possible differences between the groups were tested with one-way ANOVA (continuous variables) and Fisher's exact test (binary variables). The median with interquartile range (IQR) was presented. Kruskal-Wallis test was used to determine differences between the IHC scores of SMAD4 and SMAD7 in active CD, remission CD, and controls. Spearman's rho rank test was used for correlations between endoscopic SES-CD, histopathological score, SMAD4, and SMAD7. A two-sided *p* value < 0.05 was considered significant. For all statistics, IBM SPSS Statistics 22 (SPSS, Chicago, USA) and GraphPad Prism version 7.02 (GraphPad Software Inc., California, USA) were used.

## 3. Results

### 3.1. SMAD4 Is Markedly Downregulated in Ileal Epithelial Cells from CD Patients

**Figure 1:**
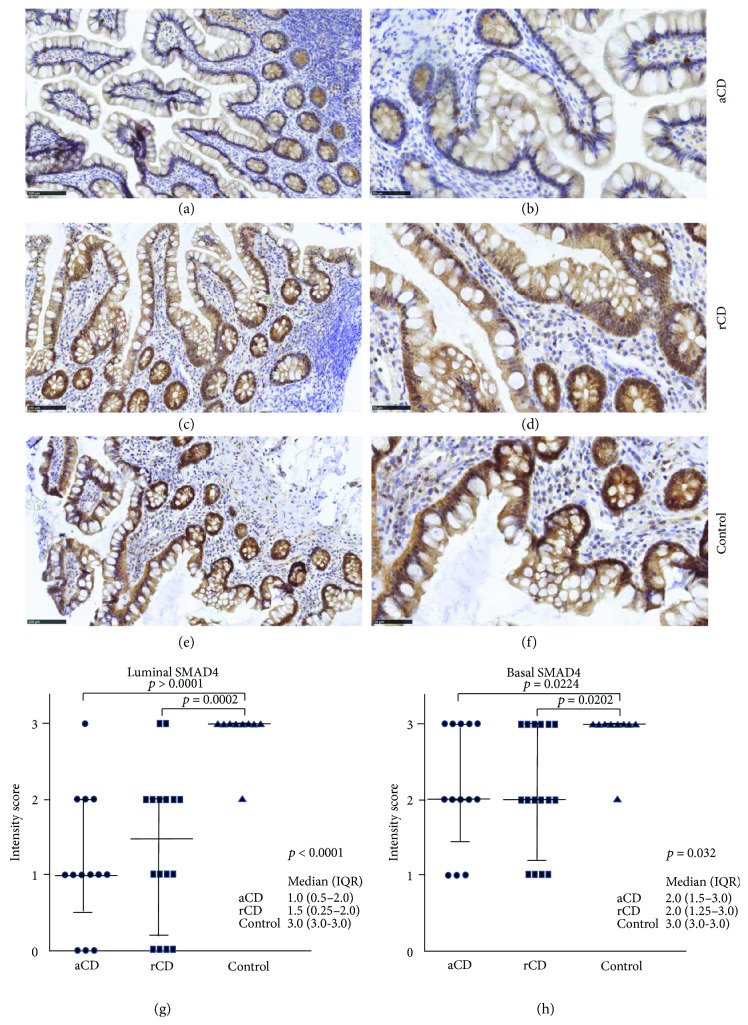
Immunohistochemical staining of SMAD4 on ileal biopsies. The luminal SMAD4 protein expression (brown color) was decreased in CD patients with active disease (aCD) (a, b) compared to CD patients in remission (rCD) (c, d) and healthy controls (e, f). Original magnification ×20 (a, c, and e) and ×40 (b, d, and f). Luminal (g) and basal (h) SMAD4 was significantly downregulated in CD patients compared to controls. Median and IQR are shown.

**Figure 2:**
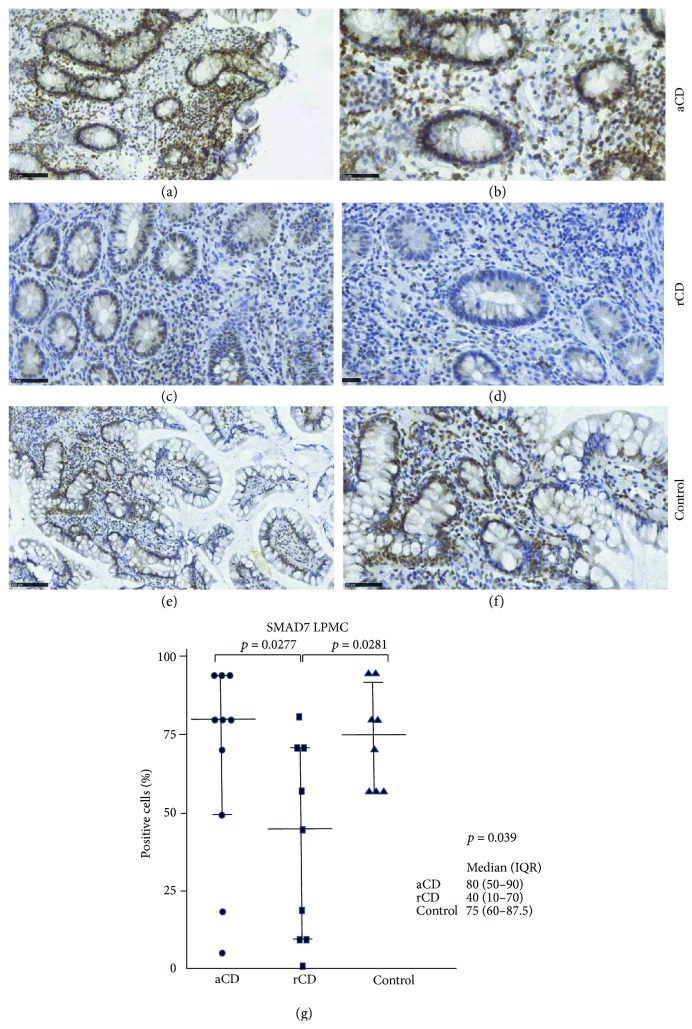
Immunohistochemical staining of SMAD7 on ileal biopsies. The lamina propria mononuclear cell (LPMC) SMAD7 protein expression (brown color) was similar in CD patients with active disease (aCD) (a, b) compared to controls (e, f). SMAD7 was downregulated in CD patients in remission (rCD) (c, d). Original magnification ×20 (a, c, and e) and ×40 (b, d, and f). SMAD downregulation in CD patients in remission was significant compared to controls and active CD (g). Median and IQR are shown.

Figure 1 shows representative SMAD4 stainings on small bowel sections from CD patients with active disease or in remission as well as from a control. In active disease (Figure 1(a)), there is a pronounced downregulation of the SMAD4 protein expression in particular on the apical epithelial surface (luminal SMAD4) compared with controls (Figure 1(c)). In contrary, SMAD4 staining is moderate in remission but still decreased compared with control (Figure 1(b)). Accordingly, the median luminal SMAD4 protein expression level was significantly decreased in sections from CD patients compared to controls (*p* < 0.0001) (Figure 1(d)). In contrast, basal SMAD4 expression was moderately but significantly downregulated in the CD patients compared to controls (*p* = 0.032) (Figure 1(e)). Notably, the strongest staining for SMAD4 in the control group was in the IECs, albeit immune cells in the lamina propria also expressed SMAD4 (Figure 1(c)). In 3 (24%) of the CD patients, luminal SMAD4 was completely absent both in the IECs and LPMCs. SMAD7 was weakly to moderately expressed in the epithelial cells in some patients but strongly expressed in the LPMCs (Figures 2(a) and 2(b)). While there was no significant upregulation of SMAD7 in patients with active disease compared with controls, CD patients in remission had a significantly lower amount of SMAD7-positive LPMCs compared with CD patients with active disease and controls (*p* = 0.039) (Figure 2(d)). There was no significant correlation neither between SMAD7 and luminal SMAD4 expression (*r*_s_ = 0.312; *p* = 0.106) nor between SMAD7 and basal SMAD4 expression (*r*_s_ = 0.210; *p* = 0.284).

### 3.2. Luminal SMAD4 but Not SMAD7 Expression Is Inversely Correlated to Disease Activity

When correlated to disease activity, we found that luminal SMAD4 expression was significantly inversely correlated to the severity of the disease in terms of SES-CD and histopathological activity. Basal SMAD4 expression was not correlated to SES-CD or histopathological activity. Moreover, SMAD7 expression did not correlate to SES-CD or histopathological activity (Table 2).

To summarize, luminal SMAD4 was downregulated in CD patients and was inversely correlated to disease activity. SMAD7 expression was not significantly changed in CD patients compared with controls but was significantly downregulated in CD patients in remission.

During a mean follow-up of 71 weeks, 15 (54%) CD patients relapsed after a mean period of 18 weeks. Seven patients with relapse and 2 in remission were examined with repeat ileocolonoscopy and collection of ileal biopsies during the follow-up period. Interestingly, we observed an increased SMAD4 expression in the two patients in long-term remission (luminal score increased to 2) while SMAD4 expression remained low in the seven patients who relapsed (luminal score 0 or 1). This observation needs to be confirmed in a larger group of patients but indicates that the SMAD4 level is dynamic and related to disease activity.

## 4. Discussion

This is an explorative and hypothesis-generating study, with the novel primary finding that ileal SMAD4 protein expression level is substantially decreased in patients with active CD. Thus, our data point to an immunosuppressive defect in CD patients, which may be reversed for patients in long-term remission. We found the strongest expression of SMAD4 in normal IECs, and the most pronounced downregulation on the apical (i.e., luminal) surface in CD patients with active disease. Sixteen out of 29 (55%) CD patients were in histopathological remission when endoscopy was performed the first time. They still exhibited low (50%) or moderate (50%) luminal SMAD4 expression. Thus, the SMAD4 defect is not reversed with medical treatment including anti-tumor necrosis factor- (TNF-) *α* antibodies, which some patients received to obtain remission (Table 1). There was no correlation between a low level of SMAD4 and the time of relapse, but follow-up biopsies in nine patients showed that the SMAD4 level was increased (luminal score 2) in the seven patients who stayed in remission but remained low (luminal score 0 or 1) in the two who relapsed. The major SMAD4 defect was shown in the luminal, fully matured IECs as well as LPMCs. The IECs are functioning not only as a simple barrier but also as the front line of defense, orchestrating the immune response due to the ability of IECs to produce a variety of molecules including cytokines [17], antimicrobial peptides [18, 19], and proteinases [20]. The consequence of an impaired TGF-*β* signaling in the IECs may influence not only repression of the immune response but also healing of ulcers, as TGF-*β* has a prominent role in wound healing [21].

We speculate that the SMAD4 decrease is an early defect in CD, and it would be interesting to pinpoint when and how early it occurs. The SMAD4 expression is regulated by several microRNAs (miRNAs) [22–26] as well as on a posttranslational level [27, 28]. A plausible explanation on how the SMAD4 level is reduced could be that the activation of toll-like receptors on the epithelial cell surface by intestinal bacteria induces a signaling cascade through nuclear factor-kappa B, which in turn upregulates a variety of miRNAs, and some of these may inhibit the production of SMAD4 protein and thus induce a sustained SMAD4 downregulation [29]. In this context, it is interesting that the existing transformed human IEC cell line models are all either SMAD4 negative or TGF-*β* receptor II negative [30]. The profound downregulation of TGF-*β* signaling in human IEC cell lines may be explained by the fact that *TGFBRII* mutations are highly represented in tumors with microsatellite instability and *SMAD4* is mutated in more than half of colorectal tumors without microsatellite instability [6].

Previous findings have demonstrated an upregulation of SMAD7 especially in the LPMCs in active CD patients [31]. We were unable to detect an increased SMAD7 expression level in CD patients with active disease compared with controls. But interestingly, we observed a significant downregulation in CD patients in remission compared with patients with active disease. These findings support the effects of mongersen therapy, as shown previously [31]. One could speculate that the lack of effect of mongersen in the phase 3 study is due to a low level of SMAD4 that is still present in CD patients with active disease. A combination of knockdown of *SMAD7* as well as induction of SMAD4 would be more efficient in the restoration of full TGF-*β* signaling.

The limitations in this study include only a small cohort was analyzed. Also, only the terminal ileum was investigated; inclusion of the colon would be an interesting study in the future. Most of the patients (69%) in remission had previous disease activity in the colon, and the control group was significantly older than the CD patients. A study with an analysis of the LPMC population, as well as inclusion of a larger number of patients in the follow-up study, is warranted. Only the semiquantitative analysis IHC was used. Quantitative analyses (quantitative reverse transcriptase polymerase chain reaction (qRT-PCR) and western blotting) have been performed. However, the material analyzed was lysates of cellular fractions (epithelial and intraepithelial cells) of the whole biopsy. We found that SMAD4 downregulation was mainly seen at the apical surface of the biopsy, which accounts for a small area of the biopsy (Supplementary Figure 1). Thus, biochemical analyses of the whole biopsy do not reflect the finding seen by IHC (data not shown). Future protocols should include qRT-PCR and proteomic analysis on laser microdissected cells.

In conclusion, we have shown that the SMAD4 protein level is markedly downregulated in particular in the mature IECs from CD patients, and this is significantly and inversely correlated with disease activity. The ileal SMAD7 protein level was downregulated in CD patients in remission. This may contribute to maintain chronic inflammation due to insufficient TGF-*β* activity.

## Figures and Tables

**Table 1:** Patient characteristics.

	Active CD	Remission CD	Controls	*p* value
Number (%)	13 (34)	16 (42)	9 (24)	
Female (%)	6 (46)	7 (44)	3 (33)	0.839
Age (median (IQR))	38 (29.00–55.50)	44 (35.75–52.75)	75 (56.50–76.50)	0.001
*Age at diagnosis*				
A1 (below 16 years) (%)	0	0	—	
A2 (17–40 years) (%)	10 (77)	12 (75)	—	
A3 (above 40 years) (%)	3 (23)	4 (25)	—	
*Location*				
L1 ileal (%)	10 (77)	4 (25)	—	
L2 colonic (%)	0	11 (69)	—	
L3 ileocolonic (%)	3 (23)	1 (6)	—	
*Behavior*				
B1 nonstricturing, nonpenetrating (%)	2 (15)	10 (63)	—	
B2 stricturing (%)	10 (77)	2 (13)	—	
B3 penetrating (%)	1 (8)	4 (25)	—	
*Previous intestinal resection related to CD*				
Small bowel (%)	0	0	—	
Colorectal (%)	0	0	—	
Small bowel and colon (%)	4 (29)	2 (10)	—	
*Disease localization of active luminal CD (SES-CD > 2)*				
Small bowel (%)	10 (77)	0	—	
Colorectal (%)	0 (0)	0	—	
Small bowel and colon (%)	3 (23)	0	—	
*Ongoing medical treatment * ^∗^				
TNF inhibitors (%)	1 (7)	9 (56)	—	
Azathioprine (%)	4 (31)	9 (56)	—	
Corticosteroids (%)	2 (14)	1 (6)	—	

CD: Crohn's disease; SES-CD: Simple Endoscopic Score for Crohn's Disease. Montreal classification was used for classification of disease behavior. ^∗^In the aCD group, 1 patient received both TNF inhibitors and azathioprine; in the rCD group, 4 patients received both TNF inhibitors and azathioprine, while 1 patient received corticosteroids and azathioprine.

**Table 2:** Correlations.

	*p* value	Correlation coefficient *r*_s_
Luminal SMAD4		
SES-CD	0.001	−0.530
Histopathological activity	0.013	−0.399
Basal SMAD4		
SES-CD	0.190	−0.217
Histopathological activity	0.281	−0.179
SMAD7		
SES-CD	0.143	0.284
Histopathological activity	0.294	0.205

Correlation of disease activity to SMAD4 and SMAD7. Spearman's rho correlation was made on the entire cohort.

## Data Availability

The data used to support the findings of this study have not been made publicly available.

## Supplementary Materials

Supplementary Materials Figure 1: immunohistochemical staining of SMAD4 on an ileal biopsy from a rCD patient. Luminal and basal part of the biopsy is separated by a black drawing.
