# Supplementary material for: SMAD4 Protein Expression Is Downregulated in Ileal Epithelial Cells from Patients with Crohn's Disease with Significant Inverse Correlation to Disease Activity
Source: Gastroenterol Res Pract. 2018 May 24;2018:9307848. doi: 10.1155/2018/9307848 (PMC5994270; doi:10.1155/2018/9307848)
Supplement: Supplementary Materials — Figure 1: immunohistochemical staining of SMAD4 on an ileal biopsy from a rCD patient. Luminal and basal part of the biopsy is separated by a black drawing. [file 9307848.f1.pdf]

Supplementary Figure 1

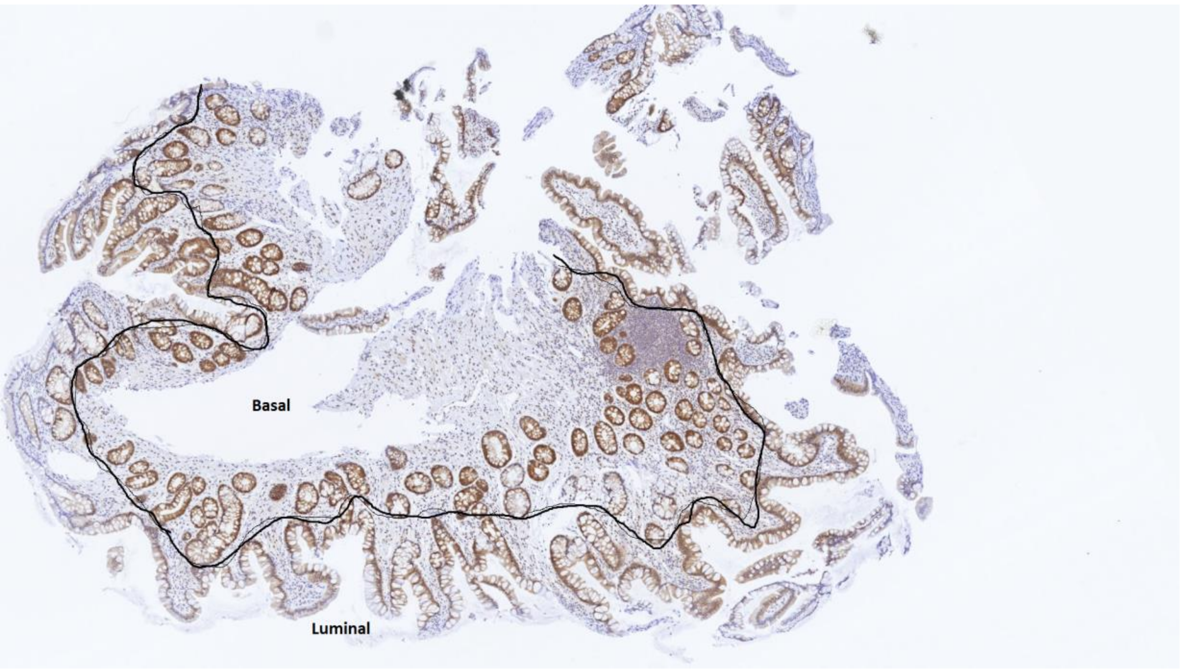

Supplementary Figure 1. Immunohistochemical staining of SMAD4 on an ileal biopsy from a rCD patient. Luminal and basal part of the biopsy is separated by a black drawing.
